# Anti-HCV RNA Aptamers Targeting the Genomic *cis*-Acting Replication Element

**DOI:** 10.3390/ph5010049

**Published:** 2011-12-28

**Authors:** Soledad Marton, Beatriz Berzal-Herranz, Eva Garmendia, Francisco J. Cueto, Alfredo Berzal-Herranz

**Affiliations:** Instituto de Parasitología y Biomedicina “López-Neyra”, IPBLN-CSIC, P.T. Ciencias de la Salud, Av. del Conocimiento s/n, Armilla, 18100 Granada, Spain; Email: smarton@ipb.csic.es (S.M.); bbh@ipb.csic.es (B.B.-H.); evagaresp@gmail.com (E.G.); fcracfc@gmail.com (F.J.C.)

**Keywords:** aptamer selection, SELEX, RNA aptamers, anti-HCV aptamers, aptamer therapeutics

## Abstract

Hepatitis C virus (HCV) replication is dependent on the existence of several highly conserved functional genomic RNA domains. The *cis*-acting replication element (CRE), located within the 3' end of the NS5B coding region of the HCV genome, has been shown essential for efficient viral replication. Its sequence and structural features determine its involvement in functional interactions with viral RNA-dependent RNA polymerase and distant RNA domains of the viral genome. This work reports the use of an *in vitro* selection strategy to select aptamer RNA molecules against the complete HCV-CRE. After six selection cycles, five potential target sites were identified within this domain. Inhibition assays using a sample of representative aptamers showed that the selected RNAs significantly inhibit the replication (>80%) of a subgenomic HCV replicon in Huh-7 cell cultures. These results highlight the potential of aptamer RNA molecules as therapeutic antiviral agents.

## 1. Introduction

Some 3% of the World’s population is infected with hepatitis C virus (HCV), the major etiological agent of non-A non-B hepatitis [1,2]. No protective vaccine is available and current therapeutic strategies based on the combination of pegylated α-interferon and ribavirin are effective in only 40% of patients [3]. The quasispecies structure of the infecting viral population, a consequence of the high mutation rate of the HCV genome, is the main factor responsible for therapeutic failure [4]. The identification of conserved viral genomic functional elements that could act as potential therapeutic targets has therefore attracted much research attention.

**Figure 1:**
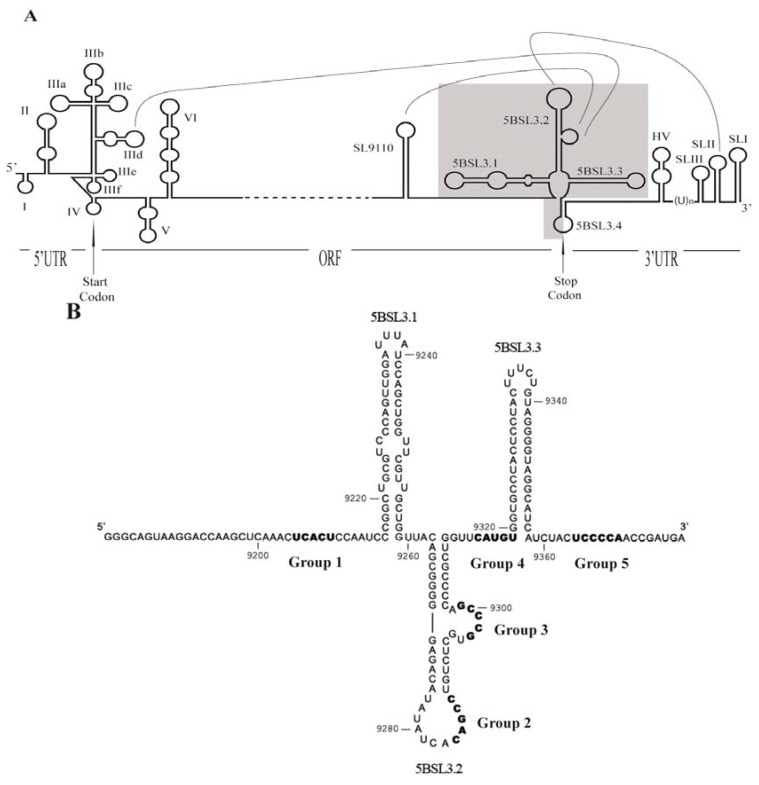
(**A**) Diagram of the secondary structural elements of the 5' and 3' ends of the HCV genome. ORF indicates the only open reading frame, which is flanked by the 5' and 3' untranslatable regions (UTR). The translation start and stop codons are indicated by an arrow. The main structural domains at the 5' end are numbered I to VI; subdomains are identified by adding a lower case letter (IIIa–IIIf). The representation of the 3' part of the genome includes (from the 3' end): The 3' X-tail region that includes the SLI, SLII and SLIII domains; the polyU stretch [(U)n]; the hypervariable region (HV); the CRE domain, which includes domains 5BSL3.1, 5BSL3.2 and 5BSL3.3 at the 3' end of the coding region; and the stem-loop SL9110. Long range RNA-RNA interactions are depicted by lines. The genomic fragment used as a target for the *in vitro* selection procedure is shaded in the diagram; (**B**) The sequence and secondary structure for the HCV-CRE_194_ genomic RNA fragment used as target. Numbering refers to the nucleotides positions of the HCV Con1 isolate [33]. Motifs complementary to the consensus sequences of the groups of selected aptamer are shown in bold.

The HCV genome is a 9,600 nt-long positive strand RNA molecule [1]. It encodes a single open reading frame (ORF) flanked at both ends by untranslatable regions (UTR) (Figure 1A) that are characterized by high sequence and secondary structure conservation rates across viral genotypes [5]. These UTRs contain domains essential for viral replication, translation and infectivity [6,7,8,9,10,11,12]. Conserved functional RNA domains have also been identified within the coding region. Of particular interest is the *cis*-acting replication element (CRE) in the 3' end of the NS5B coding region. Recognised as an important partner in viral RNA synthesis [13,14], it folds into a cruciform structure composed of three stem-loop motifs known as 5BSL3.1, 5BSL3.2 and 5BSL3.3 [14] (Figure 1B). Deletion and mutational analyses of these motifs have shown 5BSL3.1 and 5BSL3.2 to be essential in the replication of HCV subgenomic replicons [13,14,15,16]. The blockage of the 5BSL3.3 stem-loop by antisense peptide nucleic acids (PNAs) has shown this stem loop to have an important role in the initiation of minus-strand RNA synthesis [13]. The specific structural features of the CRE promote the recruitment of functional proteins, such as NS5B RNA polymerase [17], to the replication process. The CRE domain is also involved in RNA-RNA interactions with distant sequences within the HCV genome [15,18,19,20] (Figure 1A). An apical loop-apical loop interaction has been defined between 5BSL3.2 and the 3'SL2 domain in the 3'UTR that is important in viral RNA synthesis [15]. Further, 5BSL3.2 establishes, through its internal loop, two ALIL (apical loop-internal loop) interactions with structural elements located upstream in the NS5B coding sequence [18], and with the conserved apical loop of domain IIId in the internal ribosomal entry site (IRES) [20]. This last interaction is involved in the regulation of IRES-dependent translation mediated via the 3' end of the viral genome [19]. These data highlight the importance of these conserved genomic RNA domains in the modulation of essential steps of the viral cycle.

RNA molecules, and particularly aptamer RNAs, are good candidates for targeting functional viral RNA domains [21,22,23,24]. Most of the aptamer selection procedures described to date are based on the so-called SELEX (systematic evolution of ligands by exponential enrichment) strategy [25]. These procedures allow the identification of short oligonucleotides—Aptamers—that efficiently bind to a target molecule. The specificity of aptamer molecules resides in their sequence and structure, which allow them to recognize and bind to specific chemical groups in a proper conformation within their ligand molecules. In particular, binding efficiency of aptamers targeting RNA molecules resides in the recognition of the nucleotide sequences and structure of the target RNA domain. Extensive work has been performed with the aim of isolating RNA aptamers that efficiently bind to essential functional domains in viral genomes [26,27,28,29,30,31,32]. However, the use of such molecules as therapeutic agents is yet to be explored.

The present work describes the use of an *in vitro* selection strategy to isolate RNA aptamers against the HCV-CRE. This method allowed the identification of several potential target sequences within this viral domain and provided a collection of aptamer RNA molecules specific to each. To our knowledge this is the first report describing the selection of aptamers designed to target this region. The testing of the *ex vivo* functionality of a few selected aptamers confirmed their feasibility as efficient HCV inhibitors as well as the potential of the CRE as a target for new anti-HCV therapeutic strategies.

## 2. Experimental Section

### 2.1. Construction of the Initial RNA Population

The template for the synthesis of the RNA population was assembled by the annealing and extension of the 5'aptamerCRE—GCTAT*GAATTC*TAATACGACTCACTATAGGGATATTATAGTACATAAAN_30_aggtagcgaattaaagagtagtc, and the 3'aptamerCRE—CGACTGTCgactactctttaattcgctacct, using an Oligo 3400 DNA Synthesizer (Applied Biosystems, Foster City, CA, USA). The T7 promoter region is underlined, the restriction site for *Eco*R1 is shown in italics, the lower case letters indicate complementary residues, and N_30_ denotes 30 consecutive A, G, C or T nucleotides, thus providing for different RNAs. A mixture of 3 nmol of each oligonucleotide was heated at 95 °C for 2 min, followed by slow cooling to room temperature. Full, double-stranded DNA was obtained by extension for 1 h at 37 °C with 2.5 U of the Klenow fragment of DNA polymerase (Fermentas, Burlington, ON, Canada) in the presence of 2.5 mM of each dNTP. It was then PCR-amplified (7 cycles) using Taq DNA polymerase (Biotools B&M Labs, Madrid, Spain) in the presence of the primers 3' PCR-CRE (TCGACTACTCTTTAATTCGCTACCT) and 5' PCR-CRE (GCTAT*GAATTC*TAATACGACTCACTATAGGGATATTATAGTACATAAA). The resulting dsDNA was used as a template for *in vitro* transcription using the T7 RiboMAX^TM^ transcription kit (Promega, Madison, WI, USA) to yield the initial RNA population.

### 2.2. Selection of Aptamers

The isolation of active RNA molecules for binding to the viral RNA was performed in a sepharose-streptavidin column (HiTrap Streptavidin HP Columns, Amersham Biosciences GmbH, Uppsala, Sweden) loaded with biotinylated viral HCV-CRE_194_ RNA fragments. These fragments were obtained by *in vitro* transcription of the *Sal*I linearised plasmid construct pUC18-T7HCV9181-9371 [20]. The latter plasmid is constructed by cloning the 9181-9371 fragment of HCV con1 isolate of genotype 1b [33] between the *Eco*RI and *Sal*I restriction sites of the pUC18 vector (Fermentas). HCV-CRE_194_ was internally biotinylated during its synthesis by adding 188 µM of biotin-16-uridine-5'-triphosphate (biotin-16-UTP, Roche Diagnostics, Molecular Biochemicals, Mannheim, Germany) to the reaction mix. The amount of biotin-modified nucleotide added was calculated to yield approximately one biotin residue per molecule [34]. 38 µmol of the biotinylated viral RNA target were immobilised in the sepharose-streptavidin column following the manufacturer’s instructions. The column was then equilibrated with binding buffer (50 mM Sodium cacodylate, 300 mM KCl with 10 mM MgCl_2_) and stored at 4 °C until use. Prior to the initial selection step, 6.4 nmol of the RNA population (P0) were passed with binding buffer through a pre-equilibrated column lacking the biotinylated target RNA, and incubated for 30 min at 25 °C, to prevent false-positives due to non-specific binding to the column matrix. Aliquots of 4.5 nmol of the resulting RNA molecules were loaded, in 1 mL of binding buffer, onto the target RNA-prepared column and incubated for 30 min at 25 °C. Unbound molecules were discarded by washing three times with 5 mL and once with 10 mL of cacodylate buffer at 25 °C. All RNA molecules bound to the column were eluted by washing with 10 mL of binding buffer heated at 95 °C. They were then concentrated to 10 µL with Centricon YM-10 (Millipore, Bedford, MA, USA).

### 2.3. Amplification of Selected Molecules

The eluted, concentrated active RNA molecules were retrotranscribed using SuperScript^TM^ III reverse transcriptase (Invitrogen, San Diego, CA, USA) in the presence of 10 pmols of 3' PCR primer and 10 mM of each NTP for 1 h at 55 °C. The reaction was stopped by heating for 15 min at 70 °C. cDNA was then amplified with Certamp mix DNA polymerase (Biotools B&M Labs) in the presence of 25 pmol of the 3' PCR-CRE and 5' PCR-CRE primers. The resulting dsDNA product was used as a template for *in vitro* transcription using the T7 RiboMAX^TM^ transcription kit (Promega), and the resulting RNA population (P1) either subjected to a new selection round or cloned in the pGEMT-Easy vector (Promega) for sequence analysis. Selection conditions were changed between selection cycles by reducing the target:RNA pool ratio from 5:1 to 1:2 as well as increasing the incubation temperature to 37 °C in round 5, and by reducing the ionic strength from 10 mM MgCl_2_ to 2 mM MgCl_2_ in round 7. The new conditions were maintained in the following cycles.

### 2.4. Inhibition Assays of the HCV Replication

HCV replication assays were performed using a human hepatocarcinoma cell line harbouring an HCV subgenomic replicon system (Huh-7 NS3-3'; [33,35]) as previously reported [19,31]. Cell monolayers were maintained in Dulbecco’s modified Eagle’s medium (DMEM) supplemented with 20% heat-inactivated foetal bovine serum (FBS, Invitrogen) and 0.5 mg mL^−1^ G-418 at 37 °C in a 5% CO_2 _atmosphere. Twenty hours before transfections, ~80,000 cells were seeded onto a 24 well plate in DMEM supplemented with 20% FBS. Cells were transfected using TransFectin^TM^ lipid reagent (Bio-Rad) plus 3 µg of the aptamer RNA molecule, and harvested 20 h post-transfection. Viral HCV RNA was quantified by real time RT-PCR as previously described [31]. Briefly, 20 ng of total intracellular RNA extracted with Trizol (performed following the manufacturer’s instructions) were reverse-transcribed with the High Capacity cDNA Reverse Transcription Kit (Applied Biosystems). cDNA was diluted with Taqman Gene Expression Master Mix (Applied Biosystems) and amplified by PCR over 40 cycles (15 s a 94 °C and 1 min at 60 °C) with specific oligonucleotides C-149 and C-342 [36]. The fluorogenic Taqman probe (FT-275) was added to the PCR mixture to a final concentration of 150 nM [36]. Quantification of the mRNA GAPDH was performed with the Human GAPD (GAPDH) Endogenous Control kit (Applied Biosystems). Reactions were run in an ABI PRISM 7000 Sequence Detector System (Applied Biosystems). Data were analysed using ABI PRISM 7000 SDS software v.1.1 (Applied Biosystems).

## 3. Results and Discussion

### 3.1. In Vitro Selection for Aptamers Targeting the HCV-CRE

**Figure 2:**
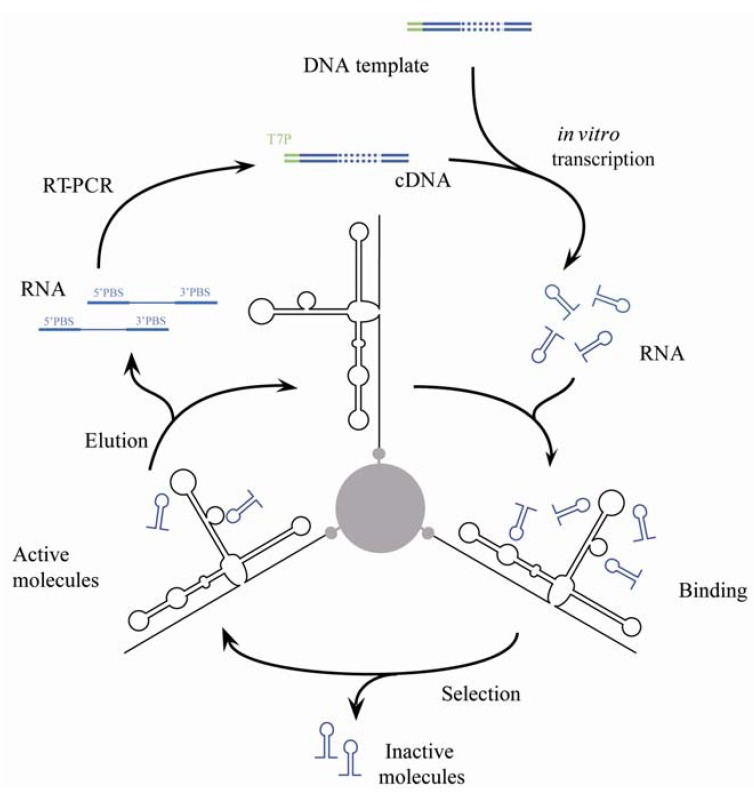
Diagram of the *in vitro* selection procedure. Details of the protocol are given in the Experimental Section. T7P, sequence promoter of the bacteriophage T7 RNA polymerase; PBS, Primer binding site.

The starting RNA population (P0) consisted of 75 nt-long RNA molecules containing a 30 nt-long variable region flanked by fixed sequences that act as primer binding sequences necessary for the amplification of active molecules (see  Experimental Section). Although the design of the initial population could have yielded a theoretical sequence heterogeneity of more than 1 × 10^18^ sequence variants, the experimental constraints limited the number to 1 × 10^15^. P0 RNA molecules were binding-challenged against the internally biotinylated HCV-CRE_194_, a 194 nt-long RNA fragment of the HCV 1b genome (Figure 1B), fixed to the sepharose-streptavidin column. Only molecules able to bind to the target viral RNA were introduced into a new selection cycle following their consecutive retrotranscription, amplification and transcription (Figure 2). This selection process was repeated for nine rounds, with the stringency of selection increased from the fifth generation by modifying the different experimental variables as described in  Experimental Section. A fraction of the amplified cDNA population resulting from generations 6–9 was used for cloning and sequence analysis.

### 3.2. Sequence Analysis of Selected RNA Molecules

**Figure 3:**
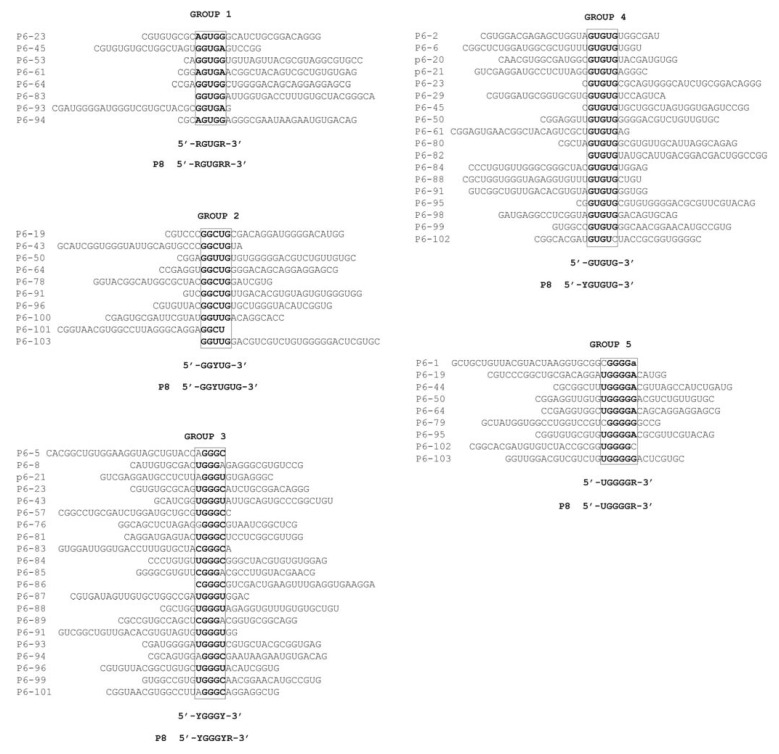
Sequence of the P6 RNA aptamers against HCV-CRE. Only sequences of the 30 nt-long random RNA motif are shown, grouped according to a common sequence shown in bold. Sequences are all aligned according to the position of the common sequence motif shown boxed. The defined consensus sequences from P6 and P8 are shown below each group. R: G or A; Y: C or U.

The molecules that bound to the HCV-CRE_194 _viral RNA after six rounds of selection (P6) were cloned. Sequence analysis of 43 clones allowed the identification of selected sequence motifs common to several molecules; these were used to classify the selected molecules into five different groups or families (Figure 3). Some selected aptamers shared more than one consensus sequence; they were therefore classifiable as members of different aptamer groups. No strong bias was observed for the localization of the consensus sequence in the variable region. Similarly, no sequence bias was observed for any other nucleotide of the variable region outside of a consensus motif. As expected, sequence analysis of the HCV-CRE_194_ sequence revealed that each of the selected consensus sequences was complementary to a defined domain within the viral target (Figure 1B). These results suggest that each of the selected consensus motifs are involved in binding the HCV-CRE_194_, with the complementary sequence within the viral RNA the most plausible target. The selected putative target sequences, ordered from 5' to 3' within the HCV-CRE sequence, were mapped to nucleotide positions 9205 to 9209 at the 5' of the 5BSL3.1 domain for aptamer group 1; to nucleotides 9284 to 9288 at the apical loop of 5BSL3.2 and 9297 to 9301 at the internal loop of 5BSL3.2 for aptamer groups 2 and 3 respectively; to nucleotides 9314 to 9318 at the linker between 5BSL3.2 and 5BSL3.3 for aptamer group 4; and to nucleotides 9364 to 9369 at the 3' of the 5BSL3.3 domain for aptamer group 5 (Figure 1B). It is interesting to note that both the apical and the internal loop of the 5BSL3.2 domain have been previously shown involved in long distant RNA-RNA interactions with other regions of the HCV genome [15,18,19,20]. The identification of both sequences as potential targets confirms this selection procedure to be a powerful tool for the identification of RNA targets prone to interact with other RNA sequences. RNA probing assays are currently being planned at our laboratory to confirm the involvement of the complementary RNA sequence motifs in viral genome-aptamer binding.

Sequence analysis of the cDNA libraries of active molecules resulting from the P7, P8 and P9 selection rounds identified no significant qualitative changes that might alter the aptamer consensus sequences arrived at in P6 (data not shown). None of the identified consensus sequences were counter selected neither new consensus motifs were selected from P7 to P9. Nevertheless, this analysis was able to identify consensus sequences extended by 1 or 2 nucleotides, and clarified nucleotides whose identity remained ambiguous in the P6 round (Figure 3). Thus, the 5 nt-long consensus sequence 5' RGUGR 3' associated with the group 1 aptamers was redefined as the 6 nt-long consensus sequence 5' RGUGRR 3' seen in the P8 population. Similarly, the 5' GGYUG 3' consensus of the group 2 aptamers became 5' GGYUGUG 3' in generation P8, and the 5' YGGGNR 3' of group 3 became 5' YGGGYR 3'. Finally, the 5' GUGUG 3' consensus sequence for the group 4 aptamers became the 6 nt-long 5' YGUGUG 3'. No changes were observed in the group 5 aptamer consensus sequence. In all cases the redefined consensus sequence matched an extension of the corresponding putative target within the viral RNA. These results indicate that the selection of aptamers and their targets had been achieved in round six, and the higher selection stringency in later rounds have allowed the definition of greater sequence requirements on the aptamer sequences.

### 3.3. Inhibition of HCV Replication by Selected Aptamers in Cell Culture

**Figure 4:**
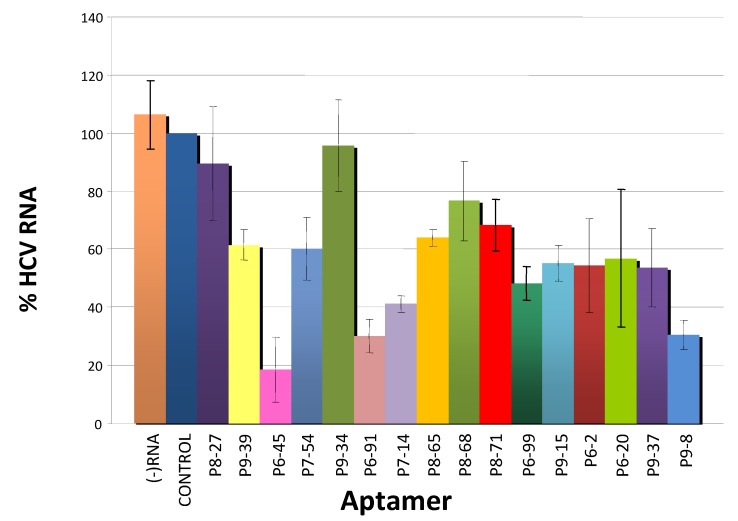
Inhibition of subgenomic HCV replicon replication in Huh-7 cells. Huh-7 cells that permit the autonomous replication of subgenomic HCV replicons, were transfected with 3 μg of the different aptamers (independently). Viral RNA was isolated and quantified as described in Experimental Section. The bar chart shows the (+) strand HCV RNA levels normalized to the value obtained with the control RNA, an 80 nt-long unrelated RNA used as an internal control. (−) RNA, cells treated with TransFectin^TM^ in the absence of any RNA. Values are the mean of at least 4 independent experiments.

To test the inhibitory activity of the selected molecules on HCV replication, and therefore to evaluate their therapeutic potential, Huh-7 cells that allow the autonomous replication of a subgenomic HCV 1b Con1 replicon were independently transfected with representative aptamers of each selected group, and the number of positive HCV strands quantified by real time RT-PCR at 20 h (see Experimental Section). Transfection with a non-related RNA of similar length was used as an internal experimental control. A total of 16 aptamers were tested (Table 1), all isolated during selection cycles P6 to P9. These aptamers represented specific groups, although some shared more than one consensus sequence. Figure 4 shows that most of these aptamers reduced the number of subgenomic replicon RNA copies (maximum 83% for aptamer P6-45) compared to those seen in control cells. The degree of inhibition exerted seems to be independent of the putative target site within the HCV-CRE. Interestingly, the most efficient inhibitors (P6-45 and P9-8) theoretically target different sequence motifs, P6-45 has two potential target sites located at the 5' of both 5BSL3.1 and 3.3 domains and P9-8 a unique site at the 3' of the CRE region. Additional experimental work is required to clearly determine the secondary structure of the aptamers and identify their target sequences, and the nucleotides involved in binding. Knowing the binding capacity of the different aptamers may help explain their inhibitory capacity. In any event, the present results demonstrate the potential of *in vitro* selection strategies for identifying RNA molecules as tools for the development of anti-HCV aptamers and to identify potential viral therapeutic targets

**Table 1:** Representative RNA aptamers assayed for their *ex vivo* inhibition of HCV replication.

Group	Aptamer	Sequence *
I (5'-RGUGRR-3'	P8-27	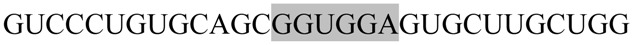
	P9-39	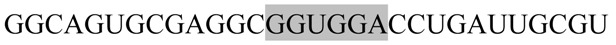
I y IV	P6-45	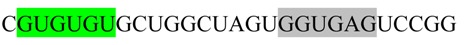
II 5'-GGYUGUG-3'	P7-54	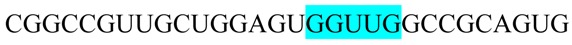
	P9-34	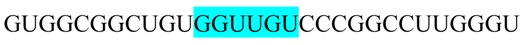
II y IV	P6-91	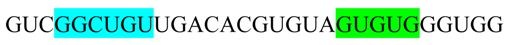
III 5'-YGGGYR-3'	P7-14	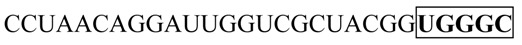
	P8-65	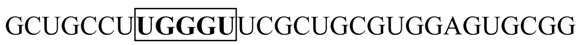
	P8-68	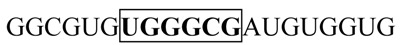
	P8-71	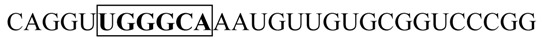
III y IV	P6-99	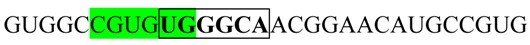
	P9-15	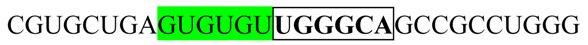
IV 5'-YGUGUG-3'	P6-2	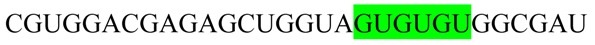
	P6-20	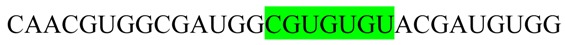
	P9-37	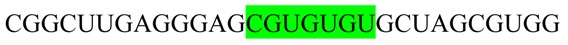
V 5'-UGGGGR-3'	P9-8	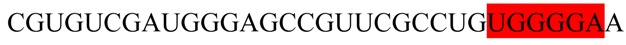

* Sequence of the variable region is shown. Common sequence is highlighted in grey for group I aptamers, in blue for group II aptamers, boxed for group III aptamers, highlighted in green for group IV aptamers and in red for group V aptamers.

## 4. Conclusions

This work reports the selection of aptamers, belonging to five different groups, which appear to interact with complementary sequences in the HCV-CRE domain. Representative aptamers showed potent (>80%) inhibition of the replication of HCV replicons, thus demonstrating their potential as antiviral agents. These results confirm the feasibility of using conserved structural RNA genomic domains as targets in new therapeutic strategies.
